# Association between pre-pregnancy body mass index and gestational weight gain on pregnancy outcomes: a cohort study in Indonesian pregnant women

**DOI:** 10.1186/s12884-022-04815-8

**Published:** 2022-06-15

**Authors:** Arif Sabta Aji, Nur Indrawaty Lipoeto, Yusrawati Yusrawati, Safarina G. Malik, Nur Aini Kusmayanti, Isman Susanto, Nur Mukhlishoh Majidah, Siti Nurunniyah, Ratih Devi Alfiana, Wahyuningsih Wahyuningsih, Karani S. Vimaleswaran

**Affiliations:** 1 Graduate School of Public Health Department, Faculty of Health Sciences, Alma Ata University, Yogyakarta, 55183 Indonesia; 2 Department of Nutrition, Faculty of Health Sciences, Alma Ata University, Yogyakarta, 55183 Indonesia; 3 Alma Ata Graduate School of Public Health, Alma Ata University, Jl Brawijaya 99, Kasihan, Bantul, Yogyakarta, 552813 Indonesia; 4 Department of Nutrition, Faculty of Medicine, Andalas University, Padang, 25127 Indonesia; 5 Department of Obstetrics and Gynaecology Department, Faculty of Medicine, Andalas University, Padang, 25127 Indonesia; 6 Eijkman Institute for Molecular Biology, Jakarta, 10430 Indonesia; 7 Faculty of Public Health, Universitas Ahmad Dahlan, Yogyakarta, Indonesia; 8 Department of Midwifery, Faculty of Health Sciences, Alma Ata University, Bantul, 55183 Indonesia; 9 Department of Nursing, Faculty of Health Sciences, Alma Ata University, Bantul, 55183 Indonesia; 10 Department of Food and Nutritional Sciences, Hugh Sinclair Unit of Human Nutrition, University of Reading, Reading, UK; 11 The Institute for Food, Nutrition, and Health (IFNH), University of Reading, Reading, UK

**Keywords:** Gestational weight gain, Pre-pregnancy BMI, Pregnancy outcomes, Overweight, Obesity, VDPM cohort study, Indonesia

## Abstract

**Background:**

Pre-pregnancy BMI (PP BMI) and gestational weight gain (GWG) are prominent anthropometric indicators for maternal nutritional status and are related to an increased risk of adverse pregnancy outcomes. This study aimed to determine the factors affecting total GWG, PP BMI and pregnancy outcomes among pregnant women in West Sumatra, Indonesia.

**Methods:**

This observational analysis was conducted among healthy women in the Vitamin D Pregnant Mother (VDPM) cohort study. A total of 195 pregnant women and their newborn babies were enrolled, and information regarding their socio-demographic characteristics, obstetric history, dietary intake and anthropometric data were assessed through direct interviews. Furthermore, the Institute of Medicine (IOM) 2009 guidelines were used to obtain the total GWG.

**Results:**

PP BMI was used to categorise the 195 pregnant women as overweight/obese (43.1%), normal (46.7%) and underweight (10.2%). There were 53.3%, 34.4% and 12.3% of women who had inadequate, adequate and excessive GWG, respectively. The multinomial logistic regression model indicated that overweight or obese women at the pre-pregnancy stage were 4.09 times more likely to have an excessive rate of GWG (AOR = 4.09, 95% CI: 1.38–12.12, *p* = 0.011) than those whose weight was normal. Furthermore, women with excessive GWG were 27.11 times more likely to have a baby with macrosomia (AOR = 27.11, 95% CI: 2.99–245.14) (*p* = 0.001) and those with inadequate GWG were 9.6 times more likely to give birth to a baby with low birth weight (LBW) (AOR = 9.60, 95% CI; 0.88–105.2) (*p* = 0.002).

**Conclusions:**

This study demonstrates that the malnutrition status prior to pregnancy and inadequate or excessive GWG status during pregnancy as significant risk factors for developing adverse pregnancy outcomes. These findings highlight the importance of providing information, preconception counselling and health education on weight management for healthy pregnancies.

**Supplementary information:**

The online version contains supplementary material available at 10.1186/s12884-022-04815-8.

## Background

Pre-pregnancy BMI (PP BMI) is a factor in a healthy pregnancy [1] and identifies when women are at risk of a difficult pregnancy due to excessive or insufficient weight gain. Obesity and being overweight during the preconception period may directly affect maternal health development [2] and adverse pregnancy outcomes such as preterm birth [3], Caesarean delivery [4], hypertensive disorder [5] and gestational diabetes mellitus [6].

Women should strive to gain the appropriate weight during pregnancy as this is critical for the growth of the fetus. However, there is a scarcity of data on the investigation of gestational weight gain (GWG) in Indonesia. As identified by the Basic Health Research Survey (Riskesdas) in 2018, the prevalence of overweight, obesity and central obesity status among the adult Indonesian population was 13.6%, 21.8% and 31%, respectively [7]. Recent research in a West Sumatran population by Soltani et al. (2017) found that 20.1% and 21.7% of the participants were underweight and overweight, respectively, while 5.3% were obese based on the Asia–Pacific BMI classifications [8, 9]. Furthermore, an Indonesian study reported that women with inadequate GWG had higher odds of giving birth to prematurity and small-for-gestational-age (SGA) infants, while those with increasing maternal BMI had higher odds of giving birth to a newborn with macrosomia [8]. The prevalence of obesity among adults in Indonesia doubled over the decade from 2007 to 2018 (10.5–21.8%), which should be considered when implementing strategies to achieve an ideal PP BMI and adequate GWG [7]. Given the importance of maternal PP BMI and GWG on pregnancy outcomes, it is vital to examine these factors in communities experiencing socio-economic transition and various nutritional statuses.

Pregnancy outcomes such as birth weight, length and head circumference are vital indicators of a newborn’s general health [10]. Low birth weight (LBW) has been found to contribute to around 60–80% of neonatal deaths [11]. Accordingly, one promising method for reducing and preventing LBW is to increase the optimum weight gain during pregnancy. A meta-analysis of 45 studies investigating the relationship between PP BMI and birth size outcomes found that a low PP BMI was associated with SGA and lower birth weight. In contrast, a high PP BMI was associated with higher birth weight or macrosomia [12]. Moreover, systematic review of 35 studies found strong evidence that large for gestational age (LGA) was associated with excessive weight gain, while SGA was associated with inadequate GWG [13]. Therefore, the objective of this study was to identify the determining factors related to total GWG and the association between PP BMI, GWG and outcomes such as birth weight, length and head circumference among pregnant women in West Sumatra, Indonesia.

## Methods

### Study design

This study used prospective data collection and subjects from the Vitamin D Pregnant Mother (VDPM) cohort study in West Sumatra [14–16]. It was conducted from July 2017 to March 2018 in private maternal clinics and hospitals in diverse regions, including different places of residence such as coastal, mountainous, urban and rural areas. Subsequently, this study aimed to determine the factors related to total GWG and the association between PP BMI and GWG with newborn anthropometric measurement outcomes. The study involved all pregnant women among the Minangkabau people, an ethnic group native to the Minangkabau Highlands of West Sumatra, Indonesia, who had attended the selected public health centres. The data were collected by trained staff with sufficient knowledge and multiple time-point analyses using data from each trimester of pregnancy. These data were acquired directly from the participants through questionnaires and included information from the women’s medical histories as provided by certified nutritionists and midwives. Further details on the data collection process are available elsewhere [17].

### The study participants

The sample size was calculated based on the VDPM cohort study, which identified the association between maternal vitamin D status and LBW [18] among 232 pregnant women from the first (T1) to the third trimester (T3) and delivery process. Eight stillbirths, 14 miscarriages and 15 participants were missing from the data collection after each trimester monitoring check. As a result, 195 pregnant women and their offspring participated in the VDPM cohort study during the data collection. All the participants gave their informed consent before the data collection process commenced.

The inclusion criteria included: 1) Minangkabau pregnant women that visited public health care facilities at each site, 2) aged 18–40, 3) currently in the first trimester of pregnancy (< 13 weeks), 4) healthy based on a general practitioner examination, and 5) willing to participate by signing the informed consent form and following the study protocol. Meanwhile, the following were excluded from the study: pregnant women with a stillbirth, abortion, congenital disabilities, pre-eclampsia, severe anaemia, hypothyroidism, those suffering from chronic diseases such as diabetes mellitus, hypertension, abnormal heart function and glandular thyroid disease, and multiple gestations. All of the participants were monitored from the first trimester to the delivery process to determine outcomes such as birth weight, length and head circumference.

### Data collection

Data characteristics were collected from the socio-demographic questionnaire, including age, working status, pregnant women’s education level and monthly household income. This study also recorded maternal characteristics such as parity status, levels of maternal nutrition knowledge and other physical activity indicators such as the duration and status of outdoor activity. Data were collected from mountainous and coastal areas. Public health centres with a high number of first-trimester pregnant women were also chosen for the data collection. The participants’ pregnancy history was obtained from the Maternal and Child Health (MCH) book, a home-based health record for pregnant women and children. The handbook can be used to monitor health, keep track of healthcare utilisation, promote maternal and neonatal education and provide information when either the pregnant woman or child is referred [19]. Data on the level of maternal nutrition knowledge were collected using a structured and validated questionnaire adapted from other studies [20]. The questionnaire contained various questions on areas such as the pregnant women’s knowledge of nutritional terms, the concept of balanced nutrition, signs and symptoms of disease, and their knowledge and practice of appropriate nutrition and food recommendations. The questionnaire consisted of 15 fixed-choice responses.

### Maternal anthropometry measurements

Pre-pregnancy body weight was collected through the participants’ interviews and reports from the pregnant women’s MCH handbook (*Buku KIA*). The MCH handbook is often considered the only record book for health workers. Each pregnant woman had an MCH handbook from the beginning of their pregnancy until the baby reached five years of age [21]. In addition, their body weight and height were measured by trained nutritionists during a monthly visit in every trimester to follow up the study and measure GWG. Body weight was determined using an electronic scale to the closest 100 g (Seca 803, Seca GmbH. Co. kg, Hamburg, Germany), while height was measured to the nearest millimetre using a stadiometer (OneMed Medicom stature meter, YF.05.05.V.A.1022, Indonesia).

PP BMI was estimated as the standard formula weight (kg) divided by the square of body height (m) using the self-reported pre-pregnancy weight [22]. Subsequently, PP BMI was classified into four categories, as shown in Table 1, according to the Asian-Pacific population cutoff points recommended by the World Health Organization (WHO) (underweight, < 18.5 kg/m^2^; normal, 18.5–22.99 kg/m^2^; overweight, 23.00–24.99 kg/m^2^; and obese ≥ 25 kg/m^2^) [9].

**Table 1:** Body mass index and gestational weight gain classifications

**PP BMI Status**	**International BMI classification in kg/m**^**2**^[23]	**Asian-Pacific BMI classification in kg/m**^**2**^[9]	**IOM-recommended GWG in kg** [24]
Underweight	< 18.50	< 18.50	12.50–18.00
Normal	18.50–24.99	18.50–22.99	11.50–16.00
Overweight	25.00–29.99	23.00–27.49	7.00–11.50
Obese	≥ 30	≥ 27.50	5.00–9.00

### Standard measures

Total GWG was calculated and compared with the IOM-recommended weight increase to determine the adequacy of weight growth during each trimester. The IOM guidelines state the following categories for recommended GWG: 12.5–18 kg for underweight, 11.5–16 kg for normal weight, 7–11.5 kg for overweight, and 5–9 kg for obese, as shown in Table 1. Total weight gain was calculated from the difference between the initial and final weight taken before delivery. Following the IOM classification and under the Asia–Pacific BMI classification by the WHO **(**Table 1**)**, the participants gained inadequate, adequate or excessive weight during pregnancy. In comparison to the International BMI classification (white Europeans), Asian populations have 3 to 5 per cent higher total body fat and a high correlation with health risks such as type 2 diabetes and cardiovascular disease [25]. This method was used to increase sensitivity when identifying the risk of adverse pregnancy outcomes in an Asian-based population, which includes Indonesia.

### Dietary intake measurements

Most pregnancy weight gain occurs in the second and third trimesters. Therefore, this study was conducted to identify the differences in dietary intake and food consumption in pregnant women during the third trimester as compared to the daily recommendations. Dietary intake data were assessed using the semi-quantitative food frequency questionnaire (SQFFQ) developed by Lipoeto et al. in 2004 and explained in the previous publication [26, 27]. This questionnaire was adapted for the dietary behaviour of the Minangkabau ethnic group and specifically designed to assess their dietary intake. In addition, daily energy and nutritional intakes were calculated and compared with the Recommended Daily Allowances (RDA) [28]. Information on the nutrient content of food items was obtained from the Nutrient Composition of Indonesian Foods and Nutrisurvey Database (Version 2007, SEAMEO-TROPMED RCCN University of Indonesia, Jakarta, Indonesia) [29]. Nutrient intakes were presented in actual grams/day, while energy was presented in kcal/day.

### Newborn anthropometry measurements

The newborns’ birth weight data were recorded in the MCH handbook using a digital baby weight scale (Seca 385, Seca GmbH. Co. kg, Hamburg, Germany), while the length was measured to the nearest millimetre using a stadiometer (OneMed Medicom stature meter, YF.05.05.V.A.1022, Jakarta, Indonesia). Newborn anthropometry status was classified according to the WHO Child Growth Standards for 1) head circumference for age: small head circumference, < 35 cm and normal head circumference, ≥ 35 cm; 2) weight for age: LBW < 2,500 g and normal birth weight ≥ 2,500 g; and 3) length for age: short birth length, < 50 cm and normal birth length, ≥ 50 cm [30]. Additionally, SGA was calculated as the weight below the 10th percentile for gestational age [31].

### Data analysis

All survey data were analysed and cleaned using IBM SPSS Statistics for Windows (version 23.0; SPSS, Inc., Chicago, IL, USA). Descriptive statistics provided basic information about the variables, with both numeric and categorical data. These data were presented as the mean levels of continuous variables as a mean ± SD, while numbers and percentages were used for the numeric and categorical data.

The association between PP BMI and pregnancy outcomes was used to identify the effects of these two variables. The indicators of pregnancy outcomes were classified into continuous and categorical data. Pregnancy outcomes in the form of continuous variables were total GWG, birth weight, birth length, head circumference, gestational age (GA) at delivery and number of antenatal care (ANC) visits, while the categorical variables were status of inadequate weight gain, spontaneous vaginal delivery, Caesarean section, low-birth-weight status, macrosomia, and SGA and LGA status. Furthermore, the independent variables were PP BMI, obstetric history, demographic and socio-economic characteristics, dietary intake and maternal nutrition knowledge, while the dependent variables were GWG status and pregnancy outcomes.

A chi-square test analysed the categorical data, and a one-way ANOVA was used to compare the dependent (total GWG status) and independent effects of known risk factors such as dietary intake, education level, socio-economic characteristics, demography, PP BMI and obstetric history. Meanwhile, a *p*-value smaller than 0.25 and other variables of known clinical relevance could also be included for further multivariable analysis. PP BMI, maternal nutrition knowledge levels and duration of outdoor activity were selected for further multivariate analysis as they had a *p*-value < 0.25. The first logistic regression models were used to estimate the odds ratio (OR) of the dependent (total GWG) and independent (PP BMI, maternal nutrition knowledge, duration of outdoor activity) variables. The second regression model was used to identify the association between one independent (PP BMI) and the dependent variables (total GWG and pregnancy outcomes). The third regression model was used to identify the association between another independent (total GWG status) and dependent variable (pregnancy outcomes). Furthermore, adjusted odds ratios (AOR) were reported for categorical outcomes, adjusted mean differences were reported for education level for women, and geographical status, maternal age and parity were selected as confounding factors. A *p*-value with a significance of less than 0.05 was considered statistically significant.

## Results

**Fig. 1:**
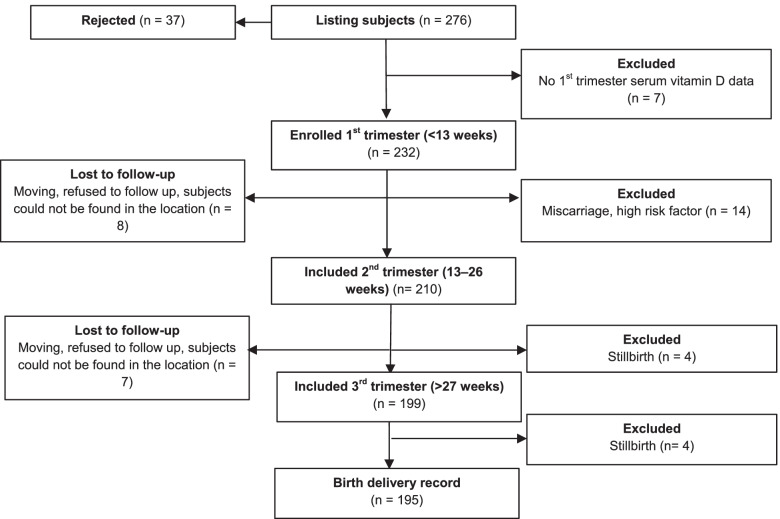
Flow chart of the VDPM cohort study

While the VDPM cohort study recruited 232 women, only 195 were eligible and included in the study sample. Therefore,195 women and their offspring were analysed after cleaning the dataset and excluding other incomplete data during the follow-up observations from the first trimester to delivery. Figure 1 presents the recruitment flow in the VDM Cohort Study in West Sumatra, Indonesia.

### Characteristics of the pregnant women

This study found that the mean age of the pregnant women was 29.7 ± 5.6 years. They were mainly in the secondary and tertiary education levels and thus relatively few of them had low levels of education, although the majority (67.7%) had no working status. About 73.8% had a monthly household income greater than or equal to the minimum wage. Most of the participants, 57.4%, engaged in no outdoor activity, while about 76.4% had a parity status of primiparous. Most of the women’s PP BMI was in the normal range, followed by the underweight category, at 46.7% and 43.1%, respectively. The majority had an inadequate GWG status compared to the GWG recommendation. The average energy intake was 2,433.5 ± 706.7 kcal/day, less than the recommended energy adequacy rate in the third trimester, which should be 2,500 kcal/day [28]. Just over half of the pregnant women, or 50.8%, had low knowledge about maternal nutrition, while only 20.5% had a high level of such knowledge. The mean gestational duration was 38.88 ± 1.91 weeks. Meanwhile, the pregnant women delivered more boy than girl infants, at 56.92% and 43.08%, respectively. Mean birth weight was in the normal range at > 2,500 g; however, head circumference and birth length were below the normal ranges at 35 cm and < 50 cm, respectively as shown in Table 2.

**Table 2:** Characteristics of pregnant women (*N* = 195)

Characteristics	Mean ± SD	n (%)
Age (years)	29.7 ± 5.6	
Energy intake (kcal/day)	2433.5 ± 706.7	
Carbohydrate intake (g/day)	309 ± 88.8	
Protein intake (g/day)	104.4 ± 40.2	
Fat intake (g/day)	87.6 ± 38.9	
Pregnant women’s education levels (n,%)		
Primary		56 (28.7)
Secondary		77 (39.5)
Tertiary		62 (31.8)
Working status (n,%)		
No		132 (67.7)
Yes		63 (32.3)
Monthly household income (n,%)		
< Minimum wage		51 (26.2)
≥ Minimum wage		144 (73.8)
Maternal nutrition knowledge levels (n,%)		
Low		99 (50.8)
Moderate		56 (28.7)
High		40 (20.5)
Outdoor activity (n,%)		
No		112 (57.4)
Yes		83 (42.6)
Duration of outdoor activity (n,%)		
< 1 h		98 (50.3)
≥ 1 h		97 (49.7)
Parity (n,%)		
Nulliparous		46 (23.6)
Primiparous		149 (76.4)
Pre-pregnancy BMI (kg/m^2^) (n,%)		
Overweight/Obese		84 (43.1)
Normal		91 (46.7)
Underweight		20 (10.2)
GWG^a^ (n,%)		
Inadequate		104 (53.3)
Adequate		67 (34.4)
Excessive		24 (12.3)
Gestational age at birth, weeks	38.88 ± 1.91	
Infant gender		
Boy		111 (56.9)
Girl		84 (43.1)
Birthweight, g	3204.87 ± 494.99	
Birth length, cm	48.56 ± 2.87	
Head circumference, cm	33.89 ± 2.52	

*BMI* Body mass index, *SD* Standard deviation, *GWG* Gestational weight gain

Data are presented as mean ± standard deviation or n (%)

^a^GWG rate was classified in line with the 2009 IOM classification recommendations based on pre-pregnancy BMI

### Factors associated with total GWG

Bivariate analysis was used to determine the factors associated with total GWG during pregnancy (Table 3). This study shows that PP BMI was significantly associated with GWG (*p*-value < 0.001). Subsequently, multinomial logistic regression indicated that women with an overweight or obese status were four times more likely to have an excessive GWG rate (AOR = 4.09, 95% CI: 1.38–12.12, *p* = 0.011) than those with normal weight status. However, this study also found that women with an overweight/obese PP BMI status had an inadequate total GWG status (AOR = 0.05, 95% CI: 0.01–0.14, *p* < 0.001), as indicated in Table 4.

**Table 3:** Factors associated with total GWG status (*N* = 195)

Variables^a^	Total GWG Status			*p*-value^2^
	**Inadequate (*n* = 104)**	**Adequate (*n* = 67)**	**Excessive (*n* = 24)**	
Age (years)	29.85 (5.9)	28.94 (5.3)	30.88 (5.3)	0.313
Energy intake (kcal/day)	2437.6 (717.4)	2480.4 (723.9)	2269.2 (600.4)	0.493
Carbohydrate intake (g/day)	306.2 (88.7)	312.3 (89.8)	311.6 (90.1)	0.905
Protein intake (g/day)	105.4 (41.9)	107.1 (40.7)	91.3 (27.4)	0.277
Fat intake (g/day)	88.7 (40.1)	90.5 (39.2)	73.1 (30.3)	0.287
Pregnant women’s education levels (n,%)				0.499
Primary	45 (43.3)	42 (62.7)	13 (54.2)	
Secondary	38 (36.5)	14 (20.9)	4 (16.7)	
Tertiary	21 (20.2)	11 (16.4)	7 (29.2)	
Working status (n,%)				0.741
No	72 (69.2)	44 (65.7)	18 (75.0)	
Yes	32 (30.8)	23 (34.3)	6 (25.0)	
Monthly household income (n,%)				0.818
< Minimum wage	28 (26.9)	18 (26.9)	5 (20.8)	
≥ Minimum wage	76 (73.1)	49 (73.1)	19 (79.2)	
Maternal nutrition knowledge levels (n,%)				0.072
Low	45 (43.3)	42 (62.7)	13 (54.7)	
Moderate	38 (36.5)	14 (20.9)	4 (16.7)	
High	21 (20.2)	11 (26.4)	7 (29.2)	
Outdoor activity (n,%)				0.824
No	58 (55.8)	39 (58.2)	15 (62.5)	
Yes	46 (44.2)	28 (41.8)	9 (37.5)	
Duration of outdoor activity (n,%)				0.193
< 1 h	48 (46.2)	34 (50.7)	16 (66.7)	
≥ 1 h	56 (53.8)	33 (49.3)	8 (33.3)	
Parity (n,%)				0.874
Nulliparous	23 (22.1)	17 (25.4)	6 (25.0)	
Primiparous	81 (77.9)	50 (74.6)	18 (75.0)	
Pre-pregnancy BMI (kg/m^2^) (n,%)				** < 0.001**
Overweight/Obese	23 (22.1)	42 (62.7)	19 (79.2)	
Normal	63 (60.6)	23 (34.3)	5 (20.8)	
Underweight	18 (17.3)	2 (3.0)	0 (0.0)	

*BMI* Body mass index, *GWG* Gestational weight gain

^a^continous variables were analysed with one-way ANOVA; categorical variables were analysed with chi-square tests

^2^*p* value significant at the *p* < 0.05 level

**Table 4:** Multinomial logistic regression model of factors associated with total GWG (*N* = 195)

Variables	Total GWG Status					
	**Inadequate**			**Excessive**		
	**OR**	**95% CI**	***p*-value**	**OR**	**95% CI**	***p*-value**
Pre-pregnancy BMI (kg/m^2^)						
Overweight/Obese	0.05	0.01–0.14	** < 0.001**	4.09	1.38–12.12	**0.011**
Normal	Ref			Ref		
Underweight	2.79	0.58–13.29	0.196	2.41	0.17–32.74	0.507
Maternal nutrition knowledge levels						
Low	0.61	0.19–1.89	0.396	0.33	0.10–1.07	0.067
High	Ref			Ref		
Moderate	1.20	0.36–4.03	0.759	0.71	0.21–2.41	0.588
Duration of outdoor activity						
< 1 h	0.97	0.41–2.26	0.946	2.30	0.89–5.97	0.085
> 1 h	Ref			Ref		

Adequate GWG as a reference group

### PP BMI and its relation to pregnancy outcomes

The association between pregnancy outcomes and PP BMI status is presented in Table 5, based on the Asia–Pacific pre-pregnancy BMI category. However, this study also contains findings regarding total GWG that incorporate the WHO international categories in Additional File 1. A significant association was identified between PP BMI status and GWG (*p* = 0.001). Women with a PP BMI < 18.5 had a GWG mean difference of -0.05 kg (95% CI: -0.38–0.29.3) compared to those with a PP BMI of 18.50–22.99 kg/m2 (normal BMI status). Overweight women with a PP BMI of 23.00 to 27.49 had a GWG mean difference of 0.55 kg (95% CI: 0.31–0.79) compared to those with normal BMI. Furthermore, the participants with an obese PP BMI status had a GWG mean difference of 1.6 kg (95% CI: 0.87–1.45). A similar result was reported in the relationship between PP BMI and birth weight outcomes, where PP BMI status was significantly associated with newborn birth weight status (*p* = 0.029).

**Table 5:** Pregnancy outcomes in relation to PP BMI

**Variables**^**b**^		**Asian-Pacific pre-pregnancy BMI category**^**a**^			***P*-value**
		** < 18.50**	**23.00–27.49**	** ≥ 27.50**	
Numbers in each category (%)		11.8	31.3	17.4	
Total GWG, kg	MD (95% CI)	-0.05 (-0.38–0.29)	0.55 (0.31–0.79)	1.16 (0.87–1.45)	**0.001**
Birth weight, g	MD (95% CI)	-184.2 (-484.3–115.9)	34.1 (-182.4–250.5)	206.9 (-53.15–466.9)	**0.029**
Birth length, g	MD (95% CI)	-0.064 (-1.83–1.70)	0.789 (-0.)	0.717 (-0.82–2.25)	0.311
Head circumference, cm	MD (95% CI)	0.1 (-1.5–1.65)	0.2 (-0.88–1.35)	0.981 (-0.36–2.32)	0.297
GA at delivery, weeks	MD (95% CI)	-0.35 (-1.53–0.83)	0.163 (-0.69–1.01)	-0.017 (-1.04–1.00)	0.753
Number of ANC visits	MD (95% CI)	0.86 (-1.02–2.74)	0.08 (-1.3–1.4)	0.7 (-0.9–2.3)	0.481
Inadequate weight gain	OR (95% CI)	1.06 (0.32–3.54)	3.57 (1.11–1.15)	1.86 (3.9–8.8)	**0.001**
Spontaneous vaginal delivery	OR (95% CI)	0.69 (0.21–2.29)	1.59 (0.75–3.39)	1.37 (0.55–3.38)	0.425
Caesarean section	OR (95% CI)	1.45 (0.44–4.81)	0.63 (0.30–1.33)	0.73 (0.30–1.81)	0.425
LBW < 2.50 kg	OR (95% CI)	1.13 (0.21–6.00)	0.83 (0.22–3.08)	0.35 (0.41–3.09)	0.351
Macrosomia > 4.0 kg	OR (95% CI)	2.40 (0)	0.74 (0.17–3.25)	1.92 (0.48–7.64)	0.204
SGA	OR (95% CI)	1.13 (0.21–6.02)	0.61 (0.15–2.56)	1.17 (0)	0.175

^a^Reference group: normal BMI 18.50–22.99 kg/m^2^ with 39.5% of participants

^b^Continous variables were analysed with linear regression; categorical variables were analysed with logistic regression

Adjusted for women’s education, geographical status, maternal age and parity

*GA* Gestational age, *BMI* Body mass index, *n* Number; *GWG *gestational weight gain, *MD *mean difference, *OR *odds ratio, *CI *confidence interval, *LBW *low birth weight, *SGA *small for gestational age

### GWG based on IOM recommendations and its relationship with pregnancy outcomes

According to recommendations from the IOM, Table 6 presents pregnancy outcomes based on the ASIA BMI classification. However, this study also conducted an analysis based on the WHO International BMI categories **(**see Additional File 2). A significant association was identified between GWG status and pregnancy outcomes such as the mean of birth weight (*p* = 0.001), head circumference (*p* = 0.029), low-birth-weight status (*p* = 0.002) and macrosomia status (*p* = 0.001). Women with inadequate GWG status gave birth to babies with a birth weight 187.3 g lower than those who had adequate weight gain. However, women with excessive GWG status had babies that weighed 208.4 g more than those with adequate status. Subsequently, women with inadequate GWG status were found to have babies with a head circumference 1.01 cm smaller than those with adequate status. Women with excessive GWG status gave birth to babies with a head circumference 0.25 cm smaller than women with adequate GWG status during pregnancy. Moreover, women with inadequate GWG status were 10.3 times more likely to have given birth to a baby with macrosomia than those with adequate status. By contrast, women with excessive GWG status were 27.11 times more likely to deliver a baby with macrosomia than those with adequate status.

**Table 6:** Pregnancy outcomes in relation to GWG according to IOM recommendations based on the ASIA BMI classification

**Variables**^**2**^		**IOM weight gain recommendation**^**1**^		***P*-value**
		**Inadequate**	**Excessive**	
Numbers in each category (%)		53.3	12.3	
Birth weight, g	MD (95% CI)	-187.3 (-364.3–10.31)	208.4 (-60.34–477.1)	**0.001**
Birth length, g	MD (95% CI)	-0.65 (-1.7–0.4)	0.58 (-1.02–2.19)	0.103
Head circumference, cm	MD (95% CI)	-1.01 (-1.93- -0.09)	-0.25 (-1.65–1.14)	**0.029**
GA at delivery, weeks	MD (95% CI)	-0.17 (-0.88–0.54)	-0.15 (-1.23–0.92)	0.849
Number of ANC visits	MD (95% CI)	-0.74 (-1.85–0.36)	0.19 (-1.53–1.91)	0.183
Spontaneous vaginal delivery	OR (95% CI)	1.82 (0.91–3.65)	1.86 (0.71–4.92)	0.177
Caesarean section	OR (95% CI)	0.55 (0.27–1.10)	0.54 (0.20–1.42)	0.177
LBW < 2.50 kg	OR (95% CI)	9.6 (0.88–105.2)	1.98 (1.987–1.989)	**0.002**
Macrosomia > 4.0 kg	OR (95% CI)	10.13 (1.19–86.16)	27.11 (2.99–245.14)	**0.001**
SGA	OR (95% CI)	0.56 (0.14–2.20)	1.26 (0)	0.1563

^1^Reference group: Adequate GWG status with 34.4% of participants

^2^Continous variables were analysed with linear regression; categorical variables were analysed with logistic regression

Adjusted for women’s education, geographical status, maternal age and parity

*GA* Gestational age, *BMI *Body mass index, *n* Number, *GWG* Gestational weight gain, *MD* Mean difference, *OR* Odds ratio, *CI* Confidence interval, *Lbw* Low birth weight, *SGA* Small for gestational age

## Discussion

PP BMI and GWG reflect maternal nutritional status before and during pregnancy. The indicators can also be used to predict fetal growth and development. This study shows that socio-economic characteristics, demography, pregnant women’s education levels, working status, dietary intake and obstetric history were not significantly associated with GWG status. However, PP BMI was significantly associated with GWG (*p* < 0.001) as a factor determining GWG status. Adverse pregnancy outcomes were associated with inadequate or excessive GWG in mothers with low or high PP BMI compared to those with normal status (for all groups). In this study, adverse outcomes such as LBW and macrosomia were associated with insufficient and excessive GWG. Our findings, if replicated in future studies, may have a significant public health impact in terms of initiating strategies to raise awareness of the importance of PP BMI and GWG in preventing adverse pregnancy outcomes.

The findings align with the results of another study from the WHO Technical Report Series that reported the prevention and management of obesity as a global epidemic. The findings indicate a significant association between PP BMI and pre-pregnancy weight. Pregnant women with excessive GWG were found to have a higher PP BMI before pregnancy than those with adequate GWG [32], and PP BMI was found to significantly affect the relationship between GWG and infant birth weight. Furthermore, pregnant women with excessive GWG have an increased risk of LGA infants, with a higher risk of an overweight/obese PP BMI than normal weight [32]. Pregnant women with PP BMI in the overweight/obese category had a 4.09 times higher risk of experiencing excessive GWG (95% CI: 1.38–12.12) than those with normal PP BMI. Pregnant women with inadequate and adequate GWG were also found to have a lower PP BMI than those with excessive GWG [33]. Overweight or obese PP BMI was found to significantly increase the risk of experiencing excessive GWG [34, 35].

Total weight gain is inversely related to maternal PP BMI. Mean total weight gain during a full-term pregnancy range between 10 and 16.7 kg in normal adult women and is less than 11 kg in those who are overweight and obese. Higher total weight gains normally occur in adolescent and thin women with twin or multiple pregnancies. In most of the studies reviewed, 47–72% of obese women gained over 5–9 kg. Total GWG during pregnancy decreased when women had a higher status of BMI classification. This study also found that those with an overweight/obese nutritional status significantly had an inadequate total GWG status. Inadequate total weight gain was also common in obese compared to non-obese women [36]. Similar findings were observed in a 2010 study by Stefanie et al., where 53%, 27% and 20% of the women were in obese classes I, II and III, respectively. As the severity of obesity increased, the mean total GWG decreased, and in comparison, to obese class I, 15.8% of the class III women lost or did not gain weight during pregnancy. Obese class I, II and III status reported proportions of no weight gain or 0 kg as 1.1%, 2.1% and 3.3%, respectively. In contrast, the median weight loss was 4.2, 4.5 and 5.2 kg, and a similar proportion of women gained weight within the recommended 5–9 kg in each obesity class, at 20.5%, 23.2% and 22.4%, respectively. However, the proportion that gained weight above the recommended GWG range decreased from 64.9% in obese class I to 45.1% in class III [37].

This study also revealed that pregnant women with excessive weight gain had a higher risk of giving birth to low weight (LBW) babies than those with adequate weight. Another study conducted by Thapa and Paneru in 2017 [38] found that the percentage of babies born with LBW was higher in pregnant women with a BMI in the overweight range than those whose BMI was in the underweight range. In conclusion, mothers with excess weight experienced an increased risk of giving birth to a baby with LBW or macrosomia. Before pregnancy, overweight and obese women with an excess of GWG had an elevated risk of macrosomia and LBW babies [39]. Excess weight gain can increase fat mass in the body and is associated with greater infant body fat [40]. Various factors contribute to the amount of weight gained, which explains some of the differences observed in the patterns among population subgroups. Other potential determinants of GWG include social and environmental factors such as culture, family and the living environment; maternal factors including genetics, ethnicity and comorbidities; and energy balance [41]. Therefore, it is crucial for women in the pre-pregnancy period to monitor their BMI status and prevent adverse outcomes [39].

Moreover, several other aspects might lead to a poor outcome when PP BMI is not carefully maintained [42]. In another study, psychological factors such as depression and anxiety were found to be more prevalent in obese women, and a positive relationship was discovered between BMI and moderate/severe depressive symptoms. Additionally, obese women tend to have eating disorders and low quality of life, particularly in terms of their physical activity [43]. This therefore relates to achieving optimum nutrition and exercise habits before conception [44]. Another possible mechanism is psychosocial factors where depression, body image dissatisfaction and social support are associated with excessive GWG [45]. Low socio-economic status, education attainment and maternal mental health during pregnancy due to sleep problems are factors in an increased risk of excessive and inadequate GWG [46, 47]. Therefore, it is expected that adherence to a quality diet will help in maintaining the ideal GWG to control macrosomia. A previous study supported these results, where women with excessive pregnancy weight gain were more likely to give birth to children with macrosomia [48]. Subsequently, obese women before pregnancy were found to have essential risk factors for various adverse fetal outcomes beyond macrosomia. Among other significant concerns is the association between excessive GWG and neonatal outcomes through a Caesarean section [41]. Another study reported that associations between excessive GWG and long-term outcomes such as being overweight or obese in childhood are relevant [49]. Several theories suggest that in utero nutrition may affect the offspring’s development of chronic diseases such as diabetes, hypertension and other metabolic disorders. While this study remains in its early stages, maternal nutrition during pregnancy may have long-term consequences for the offspring, including neurocognitive outcomes [50–52].

Prospective data collection was used, and the participants came from diverse regions of West Sumatra, including different places of residence such as coastal, mountainous, urban and rural areas. The study further used the Asia-specific BMI category to measure the association of PP BMI with pregnancy outcomes. One limitation was the small participant sample size with which to represent the West Sumatran population. By the end of the data collection, nearly 30% of the participants were lost to follow-up from those initially recruited. A further limitation was that the PP BMI data were collected during the first trimester, and the pregnant women were expected to remember their body weight. This self-reported pre-pregnancy weight was obtained from the MCH book of each mother. Therefore, using the IOM’s GWG classification was also a limitation during this study as the IOM guidelines may not be relevant for certain populations, such as Asian women. As a theoretically controllable factor that can enhance outcomes, improved maternal nutrition both before and during pregnancy would be an ideal use for this study in a low-to-middle-income country such as Indonesia. Further studies should be pursued with larger sample sizes and more comprehensive approaches highlighting the interaction of nutritional status, the food environment and socio-economic factors with pregnancy outcomes.

## Conclusions

Maternal PP BMI and total GWG were significantly related to offspring size. Women who had overweight/obese nutritional status had fourfold increase to have an excessive GWG during pregnancy as well as were twice as likely to have higher birth weight and macrosomia outcomes compared to women with lower PP BMI and GWG. In addition, women who had inadequate GWG during pregnancy had ten times increased risk of low-birth-weight outcome compared to those with adequate GWG status. These findings support the importance of improving the health care services and facilities for women of reproductive age. Additionally, they highlight the need to create preconception counselling or health education to manage weight gain and reduce the risk of adverse pregnancy outcomes due to lower PP BMI and excessive GWG.

## Supplementary information

**Additional file 1. **Additional analysis pregnancy outcomes in relation to PP BMI based on international WHO BMI category.

**Additional file 2.** Additional analysis pregnancy outcomes in relation to GWG according to IOM recommendation-based International WHO BMI classification.

## Data Availability

The datasets generated and/or analysed during the current study are not publicly available as the additional results from the study are yet to be published; however, they are available from the corresponding author on reasonable request.

## Abbreviations

T1: First trimester
T3: Third trimester
GWG: Gestational weight gain
VDPM: Vitamin D pregnant mothers
PP BMI: Pre-pregnancy body mass index
LBW: Low birth weight
IOM: Institute of Medicine
IMR: Infant mortality rate
SGA: Small for gestational age
LGA: Large for gestational age
MCH: Maternal and child health
GA: Gestational age
SQFFQ: Semi-quantitative food frequency questionnaire
RDA: Recommended dietary allowance
BMI: Body mass index
