# Supplementary material for: Association between pre-pregnancy body mass index and gestational weight gain on pregnancy outcomes: a cohort study in Indonesian pregnant women
Source: BMC Pregnancy Childbirth. 2022 Jun 15;22:492. doi: 10.1186/s12884-022-04815-8 (PMC9202216; doi:10.1186/s12884-022-04815-8)
Supplement: Supplementary file 1 — Additional file 1. Additional analysis pregnancy outcomes in relation to PP BMI based on international WHO BMI category. [file 12884_2022_4815_MOESM1_ESM.docx]

**Additional File 1.** Additional analysis pregnancy outcomes in relation to PP BMI based on international WHO BMI category

| **Variables^2^** |  | **WHO BMI category pre-pregnancy^1^** | | | | ***P*-value** |
| --- | --- | --- | --- | --- | --- | --- |
|  |  | **<18.50** | | **25.00-29.99** | **≥30** |  |
| Numbers in each category (%) |  | 11.8 | 31.3 | | 17.4 |  |
| Total GWG, kg | MD (95% CI) | -0.12  (-0.8-0.6) | -1.31  (-1.6-1) | | -0.46  (-1-0.2) | **0.127** |
| Birth weight, g | MD (95% CI) | -242  (-315--171) | 38  (-13-88) | | 209  (41-376) | **0.027** |
| Birth length, g | MD (95% CI) | -0.49  (-0.67- -0.32) | 0.43  (0.71-0.14) | | 0.29  (-0.1-0.68) | 0.672 |
| Head circumference, cm | MD (95% CI) | -0.14  (0.45-1.65) | -0.08  (-0.88-1.35) | | 1.12  (-0.36-2.32) | 0.227 |
| GA at delivery, weeks | MD (95% CI) | 1.32  (0.13-2.53) | 0.16  (0.09-0.22) | | 0.53  (0.17-0.88) | 0.373 |
| Number of antenatal cares | MD (95% CI) | 0.82  (-0.1-1.6) | 0.04  (-0.04-0.12) | | 0.68  (0.28-1.07) | 0.481 |
| Inadequate weight gain | OR (95% CI) | 1.15  (0.25-5.31) | 3.56  (8.35-1.52) | | 9.32  (1.16-7.47) | **0.000** |
| Spontaneous vaginal delivery | OR (95% CI) | 1.43  (0.44-4.61) | 0.69  (0.31-1.51) | | 1.29  (0.44-3.77) | 0.607 |
| LBW <2.50 kg | OR (95% CI) | 0.68  (0.13-3.47) | 1.36  (0.28-6.69) | | 1.67  (0.19-13.95) | 0.876 |
| SGA | OR (95% CI) | 0.68  (0.13-3.46) | 2.79  (0.34-23.09) | | -  - | 0.218 |

^1^Reference group: normal BMI 18.50-22.99 kg/m^2^ with 39.5% of subjects.

^2^continous variables were analyzed with linear regression; categorical variables were analyzed with logistic regression.

Adjusted for woman's education, geographical status, maternal age, and parity.

GA, gestational age; BMI, body mass index; n, number; GWG, gestational weight gain; MD, mean difference; OR, odds ratio; CI, confidence interval; LBW, low birth weight; SGA, small for gestational age; LGA, large for gestational age.
