# Supplementary material for: Association between pre-pregnancy body mass index and gestational weight gain on pregnancy outcomes: a cohort study in Indonesian pregnant women
Source: BMC Pregnancy Childbirth. 2022 Jun 15;22:492. doi: 10.1186/s12884-022-04815-8 (PMC9202216; doi:10.1186/s12884-022-04815-8)
Supplement: Supplementary file 2 — Additional file 2. Additional analysis pregnancy outcomes in relation to GWG according to IOM recommendation-based International WHO BMI classification. [file 12884_2022_4815_MOESM2_ESM.docx]

**Additional File 2.** Additional analysis pregnancy outcomes in relation to GWG according to IOM recommendation-based International WHO BMI classification

| **Variables^2^** |  | **IOM weight gain recommendation^1^** | | ***P* value** |
| --- | --- | --- | --- | --- |
|  |  | **Inadequate** | **Excessive** |  |
| Numbers in each category (%) |  | 53.3 | 12.3 |  |
| Birth weight, g | MD (95% CI) | -220.6  (-164.8-276.4) | 181.2  (-15.85-378.2) | **0.001** |
| Birth length, g | MD (95% CI) | -0.82  (-0.91- -0.73) | -0.58  (-1.26-0.09) | 0.205 |
| Head circumference, cm | MD (95% CI) | -0.96  (-0.96- -0.96) | -0.3  (-1.12-0.52) | **0.051** |
| GA at delivery, weeks | MD (95% CI) | -0.74  (-0.33- -1.16) | 0.04  (-0.66-0.74) | 0.288 |
| Number of antenatal cares | MD (95% CI) | -1.26  (-1.05- -1.49) | -0.76  (-2.02-0.49) | **0.030** |
| Spontaneous vaginal delivery | OR (95% CI) | 1.04  (0.51-2.12) | 0.57  (0.16-2.02) | 0.627 |
| LBW <2.50 kg | OR (95% CI) | 0.97  (0.28-3.30) | - | 0.394 |
| SGA | OR (95% CI) | 0.47  (0.09-2.24) | - | 0.273 |

^1^Reference group: Adequate GWG status with 34.4% of subjects.

^2^Continous variables were analyzed with linear regression; categorical variables were analyzed with logistic regression.

Adjusted for woman's education, geographical status, maternal age, and parity.

GA, gestational age; BMI, body mass index; n, number; GWG, gestational weight gain; MD, mean difference; OR, odds ratio; CI, confidence interval; LBW, low birth weight; SGA, small for gestational age; LGA, large for gestational age.
